# Prevention of severe infectious complications after colorectal surgery using oral non-absorbable antimicrobial prophylaxis: results of a multicenter randomized placebo-controlled clinical trial

**DOI:** 10.1186/s13756-020-00745-2

**Published:** 2020-06-15

**Authors:** Tessa Mulder, Marjolein Kluytmans-van den Bergh, Bart Vlaminckx, Daphne Roos, Anne Marie de Smet, Robert de Vos tot Nederveen Cappel, Paul Verheijen, Alexandra Brandt, Anke Smits, Eric van der Vorm, Erik Bathoorn, Boudewijn van Etten, Jacobien Veenemans, Annemarie Weersink, Margreet Vos, Nils van ’t Veer, Stavros Nikolakopoulos, Marc Bonten, Jan Kluytmans

**Affiliations:** 1 Julius Center for Health Sciences and Primary Care, University Medical Center Utrecht, Utrecht University, Utrecht, The Netherlands; 2 Amphia Academy Infectious Disease Foundation, Amphia Hospital, Breda, The Netherlands; 3 Department of Infection Control, Amphia Hospital, Breda, The Netherlands; 4 Department of Medical Microbiology, St. Antonius Hospital, Nieuwegein, the Netherlands; 5 Department of Surgery, Reinier de Graaf Gasthuis, Delft, The Netherlands; 6 Department of Intensive Care Medicine, University Medical Center Groningen, University of Groningen, Groningen, The Netherlands; 7 Department of Surgery, Admiraal de Ruyter Hospital, Goes, The Netherlands; 8 Department of Surgery, Meander Medical Center, Amersfoort, The Netherlands; 9 Department of Surgery, Erasmus Medical Center, Rotterdam, The Netherlands; 10 Department of Surgery, St. Antonius Hospital, Nieuwegein, The Netherlands; 11 Department of Medical Microbiology, Reinier de Graaf Gasthuis, Delft, The Netherlands; 12 Department of Medical Microbiology, University Medical Center Groningen, University of Groningen, Groningen, The Netherlands; 13 Department of Surgery, University Medical Center Groningen, Groningen, The Netherlands; 14 Department of Medical Microbiology, Admiraal de Ruyter Hospital, Goes, the Netherlands; 15 Department of Medical Microbiology, Meander Medical Center, Amersfoort, The Netherlands; 16 Department of Microbiology and Infectious Diseases, Erasmus Medical Center, Rotterdam, The Netherlands; 17 Department of Clinical Pharmacy, Amphia Hospital, Breda, The Netherlands; 18 Department of Medical Microbiology, Utrecht University Medical Center, Utrecht University, Utrecht, The Netherlands

**Keywords:** Infection control, Preoperative oral antibiotic prophylaxis, Colorectal surgery, Surgical site infection

## Abstract

**Background:**

Surgical site infections (SSIs) are common complications after colorectal surgery. Oral non-absorbable antibiotic prophylaxis (OAP) can be administered preoperatively to reduce the risk of SSIs. Its efficacy without simultaneous mechanical cleaning is unknown.

**Methods:**

The Precaution trial was a double-blind, placebo-controlled randomized clinical trial conducted in six Dutch hospitals. Adult patients who underwent elective colorectal surgery were randomized to receive either a three-day course of preoperative OAP with tobramycin and colistin or placebo. The primary composite endpoint was the incidence of deep SSI or mortality within 30 days after surgery. Secondary endpoints included both infectious and non-infectious complications at 30 days and six months after surgery.

**Results:**

The study was prematurely ended due to the loss of clinical equipoise. At that time, 39 patients had been randomized to active OAP and 39 to placebo, which reflected 8.1% of the initially pursued sample size. Nine (11.5%) patients developed the primary outcome, of whom four had been randomized to OAP (4/39; 10.3%) and five to placebo (5/39; 12.8%). This corresponds to a risk ratio in the intention-to-treat analysis of 0.80 (95% confidence interval (CI) 0.23–2.78). In the per-protocol analysis, the relative risk was 0.64 (95% CI 0.12–3.46).

**Conclusions:**

Observational data emerging during the study provided new evidence for the effectiveness of OAP that changed both the clinical and medical ethical landscape for infection prevention in colorectal surgery. We therefore consider it unethical to continue randomizing patients to placebo. We recommend the implementation of OAP in clinical practice and continuing monitoring of infection rates and antibiotic susceptibilities.

**Trial registration:**

The PreCaution trial is registered in the Netherlands Trial Register under NL5932 (previously: NTR6113) as well as in the EudraCT register under 2015–005736-17.

## Background

Surgical site infections (SSIs) are among the most common healthcare-associated infections and affect approximately 10 in every 100 patients who undergo colorectal surgery [1, 2]. SSIs were associated with a substantial increase in morbidity [3] and mortality [4, 5], prolongation of hospital stays [6, 7] and higher health-care costs [8–11]. Despite the widespread adoption of infection prevention measures aimed at reducing SSIs, the risk remains high, which underlines the importance of exploring additional precautions [2]. In the past, preoperative oral non-absorbable antibiotics were applied as an infection control strategy for colorectal surgery. Because it was assumed that local antibiotics could only be effective in an “empty” colon, simultaneous cleansing was applied with osmotic fluids [12]. Routine use of this cleansing, also referred to as mechanical bowel preparation (MBP), has recently become controversial due to lack of evidence for advantageous effects. At the same time, there are certain disadvantages, like the risk of dehydration, anastomotic leakage, or patient discomfort [13, 14]. At the same time, the oral antibiotics, which were often considered to be part of the MBP bundle, were abandoned even though their efficacy without simultaneous MBP is unclear. Our study aimed to determine the efficacy of preoperative oral non-absorbable antibiotic prophylaxis (OAP) without the routine administration of MBP on the risk of SSIs after elective colorectal surgery.

## Methods

An in-depth description of the rationale and methods was published previously [15]. The trial is registered in the Netherlands Trial Register under NL5932.

### Trial design, participants and randomization

The study was designed as a double-blind placebo-controlled randomized trial and was conducted from April 2017 through August 2018 in six Dutch hospitals. (Supplementary Table 1) Patients who were scheduled for colorectal surgery and who had no absolute contraindication for the study medication [15] were eligible to participate. Written informed consent was obtained from all participants. Eligible patients were randomly assigned in a 1:1 ratio to active OAP or placebo. The randomization was performed by an independent pharmacist, using a permuted block design with varying block sizes and stratified per study center. The study’s medication was packed in identical containers that were sequentially numbered with unique numbers. The list that linked these unique numbers to the treatment allocation was securely kept at the coordinating pharmacy (Amphia Hospital, Breda, the Netherlands). Everyone who was involved in the study was blinded to the allocation until the end of the study.

### Intervention

OAP was a solution of tobramycin (16 mg/mL) and colistin sulphate (20 mg/mL) that was taken four times daily during the three days before surgery. Each dose was 5 mL. Placebo had an identical color, smell, and taste. The study medication was packed in bottles (100 mL) and distributed with 5 mL syringes. The bottles were returned to the hospital after the intervention period and were weighted to estimate treatment compliance. All patients received perioperative intravenous antibiotic prophylaxis according to the national guidelines [16].

### Outcomes and safety reporting

**Fig. 1:**
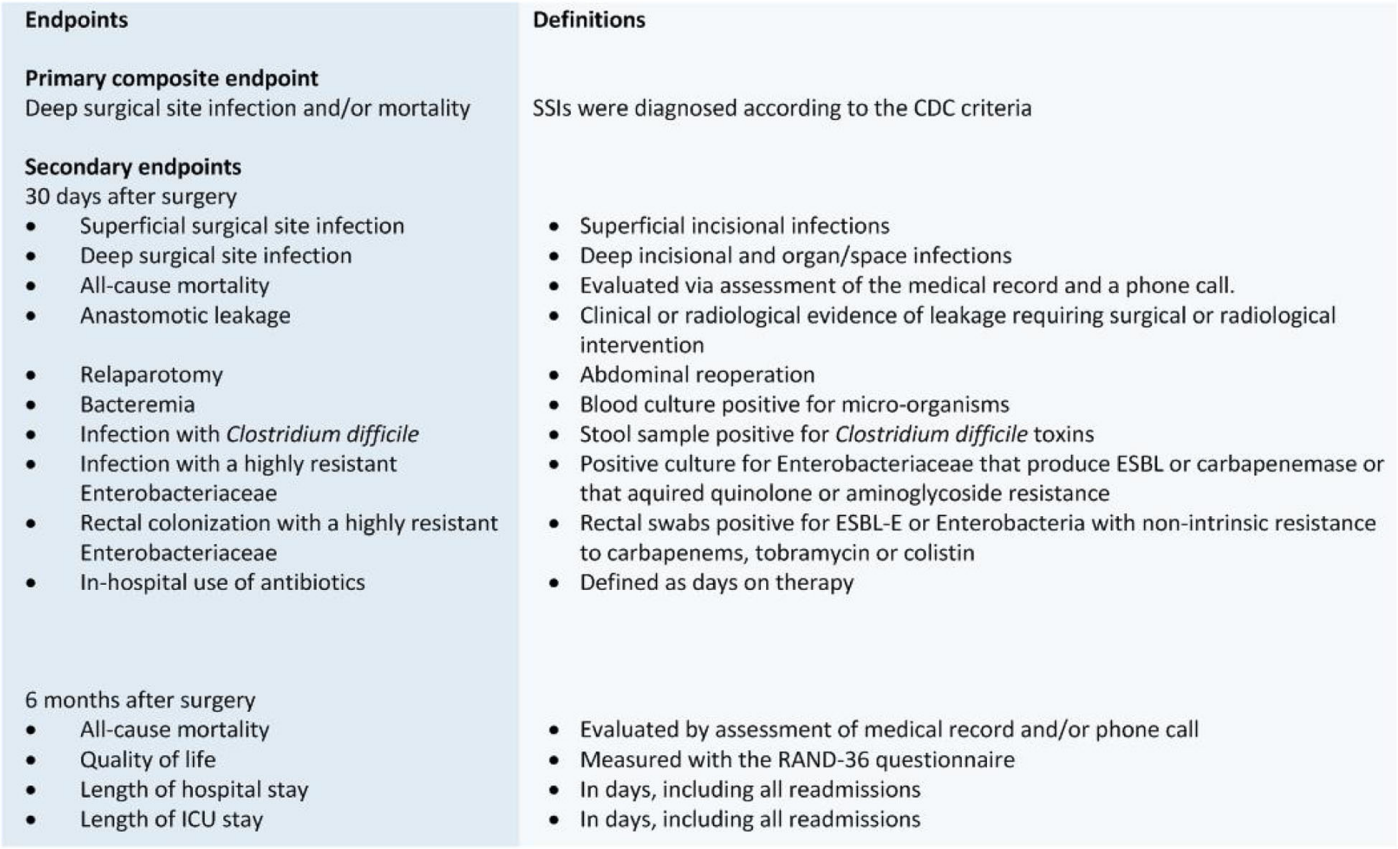
Definitions of primary and secondary endpoints. SSIs were diagnosed with the CDC criteria [17]. CDC, Centers for Disease Control and Infection Prevention; ESBL-E, extended spectrum beta-lactamase producing Enterobacteriaceae; ICU, intensive care unit; SSI, surgical site infection

Definitions of all outcomes are summarized in Fig. 1 and were described in more detail in the trial protocol [15]. The primary outcome was deep SSI and/or mortality in the 30 days after surgery. The CDC criteria were used to diagnose SSIs [17]. Rectal carriage of HRE comprised extended-spectrum beta-lactamase-producing Enterobacteriaceae (ESBL-E), and (non-intrinsic) carbapenem-resistant, tobramycin-resistant and (non-intrinsic) colistin-resistant Gram-negative Enterobacteriaceae. HRE carriage was assessed by selective screening of rectal swabs that were obtained at inclusion and 30 days after surgery. EUCAST clinical breakpoints were used to interpret MICs [18]. Cultures with a transport time of more than 72 h were excluded from analyses as reliability and quality could not be guaranteed. Quality of life was assessed with the Rand-36 questionnaire [19]. This standardized questionnaire contains eleven questions to assess the quality of life on nine different scales. The scale scores range from 0 to 100%. Adverse events (AE) related to the study medication were self-reported in a medication diary. Other protocol related AE, Serious Adverse Events (SAE), Serious Adverse Reactions (SARs), and Suspected Unexpected Serious Adverse Reactions (SUSARs) were reported according to Good Clinical Practice guidelines [20].

### Study procedures and data collection

An overview of the study procedures is provided in Supplementary Table 2. Demographic patient data, surgery characteristics, and data on the primary and secondary endpoints were collected from the medical records. Whole-genome sequencing was performed of all resistant isolates to identify the presence of acquired resistance genes.

### Statistical analysis

#### Sample size calculation

We assumed a 14.4% baseline incidence and a 40% relative reduction in the primary endpoint to calculate the sample size. This was based on results from a before-after study that was performed in a Dutch teaching hospital where OAP was introduced as a standard of care before elective colorectal surgery [21]. With a one-sided alpha of 2.5%, power of 80%, and one interim analysis, the final sample size resulted in 966 patients.

#### Data analysis

Data were analyzed according to the intention-to-treat principle. We calculated crude risks for every outcome and a corresponding risk ratio (RR) and 95% confidence interval (CI) to compare the risks in the intervention arm with the placebo arm. A per-protocol analysis was performed in the 100% compliant population. Continuous outcomes were analyzed using Student’s t-test or Mann Whitney U-test, as appropriate. Quality of life after six months was corrected for the baseline scores by calculating the change (delta) in scores. Negative deltas reflect a worse perception of quality of life compared to baseline, whereas positive values reflect improvement. We evaluated whether our study population was a representative sample of the patient population by comparing average baseline characteristics with surveillance data from a Dutch hospital that did not participate in the study. Statistical analyses were performed using R version 3.3.2.

## Results

**Fig. 2:**
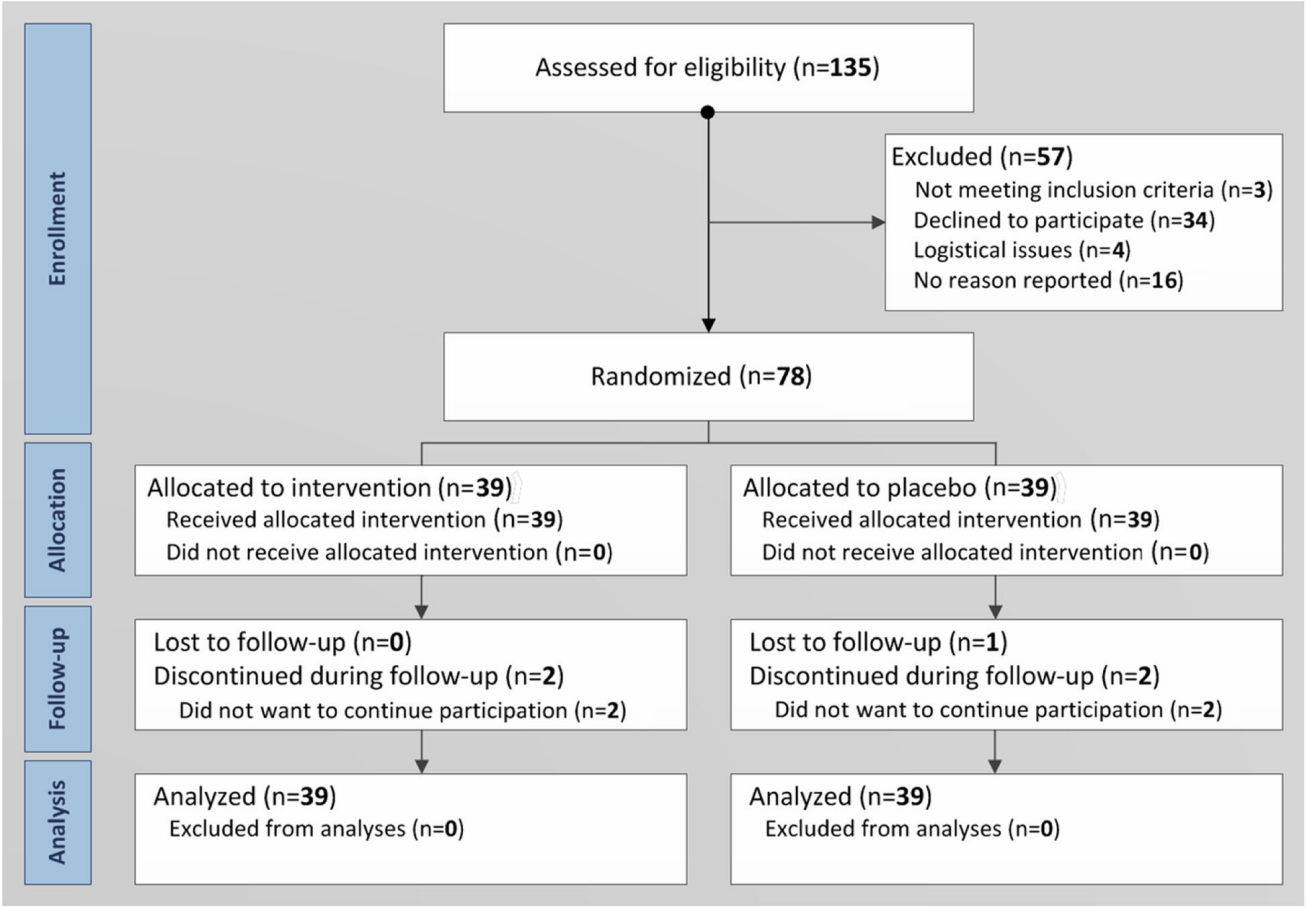
CONSORT flowchart of enrolment of participants. Logistical issues were unexpected changes in the date of surgery that led to insufficient time to complete the three-day intervention period (*n* = 1) or missed appointments for the informed consent procedure due last-minute changes in the outpatient clinic schedule (*n* = 3)

Patient enrollment is shown in Fig. 2. The number of participants and the inclusion period per hospital are presented in Supplementary Table 3. The trial ended after 18 months when 78 participants (8.1% of the sample size) had been enrolled. All patients completed the intervention period. During the six-month follow-up period, one person was lost to follow-up and four discontinued active participation but gave consent to continue data collection from their medical records.

The baseline characteristics of the participants are shown in Table 1. Thirty-nine patients were included in each treatment arm. The median age was 68 years, and 68% of patients were male. Colorectal malignancies were the indication for surgery in all except one of the patients (98.7%). Even though it was not part of routine care, MBP was applied in 3.8% of the patients. Based on the leftovers of study medication that were returned, we estimated that 57.7% of the patients took all twelve doses of study treatment.

**Table 1:** Baseline characteristics

	OAP (*N* = 39)	Placebo (*N* = 39)
Age in years	67 (61–72)	69 (61–73)
Male sex	28/39 (71.8)	25/39 (64.1)
ASA classification ≤2	26/38 (68.4)	30/36 (83.3)
Charlson Comorbidity Index		
1–2	22/39 (56.4)	25/39 (64.1)
3–4	9/39 (23.1)	2/39 (5.2)
≥ 5	8/39 (20.5)	12/39 (30.1)
Immunosuppressive therapy ^a^	0/39 (0.0)	2/39 (5.2)
BMI in kg/m^3^, *median (IQR)*	28 (24–31)	26 (23–29)
Obese (BMI > 30)	9/39 (23.1)	5/38 (13.2)
Abdominal surgery in the previous year	1/39 (2.3)	2/39 (5.2)
Oral mechanical bowel preparation	1/39 (2.3)	2/39 (5.2)
Indication for surgery		
Colorectal malignancy	38/39 (97.4)	39/39 (100)
Inflammatory bowel disease	1/39 (2.3)	0/39 (0.0)
Wound class		
Clean-contaminated (class 2)	37/39 (94.9)	39/39 (100)
Contaminated (class 3)	2/39 (5.1)	0/39 (0.0)
Type of resection		
Right sided hemicolectomy	13/39 (33.3)	9/39 (23.1)
Left sided hemicolectomy	2/39 (5.2)	4/39 (10.3)
Sigmoid resection	10/39 (25.6)	8/39 (20.5)
Low anterior resection or rectum amputation	10/39 (25.6)	15/39 (38.5)
(Sub) total colectomy	2/39 (5.2)	0/39 (0.0)
Other	2/39 (5.2)	3/39 (7.7)
Surgical approach		
Laparotomy	4/39 (10.3)	4/39 (10.3)
Laparoscopy ^b^	28/39 (72.0)	26/39 (66.7)
Robotic laparoscopy	7/39 (17.8)	9/39 (23.1)
Duration of surgery in minutes, *median (IQR)*	148 (117–185)	150 (116–215)
Duration >75th percentile ^c^	8/38 (21.1)	13/37 (35.1)
Normothermia after procedure	22/28 (78.6)	25/31 (80.1)
Stoma	6/38 (15.8)	14/39 (35.9)
Perioperative intravenous antibiotic prophylaxis	37/39 (94.9)	37/38 (97.3)
Complete compliance to study medication (all 12 doses)	23/33 (69.7)	22/33 (66.7)

Data are presented as n/N with data (%), unless specified otherwise. ASA, American Society of Anesthesiologists; BMI, body mass index; IQR, interquartile range; OAP, oral antibiotic prophylaxis

^a^ Because of chemotherapy

^b^ Of which 6 (15.4%) were converted to open procedures in OAP arm and 1 (2.3%) in placebo arm

^c^ 75th percentiles of duration of surgery, based on type of resection and approach [22]

The effect of OAP on primary and secondary outcomes is presented in Table 2. In total, nine (11.5%) patients developed outcome deep surgical site infection; all survived. Four received OAP (4/39; 10.3%) and five placebo (5/39; 12.8%). This corresponds to a risk ratio in the intention to treat analysis of 0.80 (95% CI [0.23–2.78]). There was no statistical difference between the treatment arms for any of the outcomes, except for a difference in the quality of life after six months that was improved compared to baseline on most scales in patients who had received OAP, and worsened in patients who had received placebo. In the per-protocol analysis, the risk ratio for the primary outcome was 0.64 (95% CI 0.12–3.46). The predictive power for the planned sample size given the observed results (that is, the probability of having a significant result at the end of the study, was for it to be completed, given the observed results in the 78 patients) was 67%. Due to insufficient power, we were unable to perform any of the preplanned subgroup analyses [15].

**Table 2:** Intention-to-treat analysis of OAP on the risk on primary and secondary outcomes

	OAP	Placebo	
	n/N (%)		RR (95% CI)
Deep SSI and/or mortality	4/39 (10.3)	5/39 (12.8)	0.80 (0.23–2.78)
Deep SSI	4/39 (10.3)	5/39 (12.8)	0.80 (0.23–2.78)
30-day mortality	0/39 (0.0)	0/39 (0.0)	N/A
Superficial SSI	1/39 (2.6)	1/39 (2.6)	1.00 (0.06–15.40)
Anastomotic leakage	1/39 (2.6)	2/39 (5.2)	0.50 (0.05–5.29)
Re-operation	3/39 (7.9)	2/39 (5.3)	1.50 (0.27–8.49)
Bacteremia	0/39 (0.0)	0/39 (0.0)	N/A
Infection with HRE	0/39 (0.0)	0/39 (0.0)	N/A
Infection with *Clostridium difficile*	0/39 (0.0)	0/39 (0.0)	N/A
6-month mortality	0/39 (0.0)	0/39 (0.0)	N/A
	**Median (IQR)**		***P* value**
In-hospital use of antibiotics, DOT^a^	0.0 (0.0–0.0)	0.0 (0.0–4.0)	1.000
Length of stay, days^b^	7.0 (5.0–13.0)	6.0 (5.0–12.0)	0.497
Length of ICU stay, days^b^	4.0 ^c^	0	N/A
	**Median delta (IQR)**		***P*value**
Quality of life^d^			
Physical functioning	−5.0 (−15.0–5.0)	−10.0 (−20.0–5.0)	0.124
Social role functioning	0.0 (0.0–12.5)	−12.5 (−25.0–0.0)	0.007
Physical role functioning	0.0 (0.0–25.0)	−12.5 (−93.7–0.0)	0.007
Emotional role functioning	0.0 (0.0–0.0)	0.0 (−25.0–0.0)	0.237
Mental health	4.0 (−1.0–13.0)	0.0 (−7.0–8.0)	0.072
Vitality	5.0 (−5.0–10.0)	−10.0 (−20.0–0.0)	0.002
Pain	0.0(−10.2–10.7)	−11.2 (−23.0–6.1)	0.002
General health perception	7.5 (0.0–15.0)	−5.0 (− 15.0–3.75)	0.014
Change in health	0.0 (0.0–50.0)	0.0 (−25.0–0.0)	0.092

Length of (ICU) stay, quality of life and 6-month mortality were assessed 6 months after surgery, all other outcomes were evaluated 30 days after surgery. DOT, days on therapy; HRE, highly resistant Enterobacteriaceae; ICU, intensive care unit; IQR, interquartile range; N/A, not available; OAP, oral antibiotic prophylaxis; RR, risk ratio; sd, standard deviation; SSI, surgical site infections

^a^9 patients in the OAP arm and 10 patients in the placebo arm were treated with antibiotics

^b^Including all readmissions within 6 months

^c^Only 1 patient was admitted to the ICU

^d^Delta was calculated by subtracting baseline scores from scores at 6-month follow-up. Negative delta’s reflect worse perception of quality of life on compared to baseline. Number of completed follow-up questionnaires: 27 (69.2%) OAP, 32 (82.1%) placebo

We collected 66 valid baseline rectal swabs and 62 valid follow-up rectal swabs (Table 3). There is no difference between the carriage of antibiotic-resistant micro-organisms between the two treatment arms at baseline or 30 days after surgery. In colistin-resistant isolates, no acquired colistin resistance genes were found. The carriage of tobramycin resistant species was approximately 40% both at baseline and 30 days after surgery, which was due to acquired tobramycin resistance genes in 9% of the cultures at baseline and 19.4% 30 days after surgery.

**Table 3:** Rectal carriage of (non-intrinsic) antibiotic resistant microorganisms

	Patients, n/N (%)		
	OAP	Placebo	*P* value
**Baseline**			
Number of valid rectal cultures	35/39 (89.7)	31/39 (79.5)	
Rectal carriage of HRE			
ESBL-E	2/35 (5.7)	1/31 (3.2)	1.000
Carbapenem-resistant Gram-negative bacteria	1/35 (2.9)	2/31 (6.5)	0.597
Carbapenemase-gene present	1/35 (2.9)	2/31 (6.5)	0.597
Tobramycin-resistant Gram-negative bacteria	12/35 (34.3)	14/31 (45.2)	0.452
Acquired aminoglycoside resistance gene present	1/35 (2.8)	5/31 (16.1)	0.088
Colistin-resistant Gram-negative bacteria	7/35 (20.0)	6/31 (19.4)	1.000
Acquired colistin resistance gene present	0/35 (0.0)	0/31 (0/0)	0
**30 days after surgery**			
Number of valid rectal cultures	34/36 (94.4)	28/35 (80.0)	
Rectal carriage of HRE			
ESBL-E	1/34 (2.9)	1/28 (3.6)	1.000
Carbapenem-resistant Gram-negative bacteria	0/34 (0.0)	1/28 (3.6)	0.452
Carbapenemase gene present	0/34 (0.0)	1/28 (3.6)	0.452
Tobramycin-resistant Gram-negative bacteria	15/34 (44.1)	10/28 (35.7)	0.606
Acquired aminoglycoside resistance gene present	7/34 (2.1)	5/28 (17.9)	0.318
Colistin-resistant Gram-negative bacteria	4/34 (11.9)	4/28 (14.3)	0.320
Acquired colistin resistance gene present	0/34 (0.0)	0/28 (0.0)	1.000

Data are presented as n/N (%). *P* values are estimated using Fisher’s exact test. ESBL-E, Extended-Spectrum Beta Lactamase-producing Enterobacteriaceae; OAP, oral antibiotic prophylaxis

Of the eleven SSI that developed, three were confirmed with a microbiological culture, of which two were performed on abdominal pus collected during reoperation and one directly on the incision. (Supplementary Table 4). None of the pathogens cultured were resistant to tobramycin or colistin.

Adverse events during the intervention period are presented in Table 4. Out of the 65 (83.3%) patients who returned their medication diary, 56 (86.2%) patients took at least one dose of study medication. Of those, 24 (42.9%) did not report any side effects. The most adverse events were gastrointestinal side effects. Patients who received OAP more often reported diarrhea compared to those who received a placebo (51.9% versus 20.7%) as well as nausea (11.1% versus none). During the study, there was one SAE, which was a transient ischemic attack that occurred before the start of the intervention phase. No other adverse events related to either study medication or other study procedures were reported.

**Table 4:** Adverse events

	Patients, n/N (%)		
	OAP	Placebo	*P* value
Adverse events related to study medication			
Self-reported side effects during intervention period^a^			
No side effects	13/27 (48.1)	11/29 (37.9)	0.596
Gastro-intestinal side effects			
Diarrhea	14/27 (51.9)	6/29 (20.7)	0.015
Nausea	3/27 (11.1)	0/29	0.065
Stomach ache	7/27 (25.9)	6/29 (20.7)	0.624
Loss of appetite	1/27 (3.7)	0/29	0.296
Flatulence	1/27 (3.7)	3/29 (10.3)	0.335
Other side effects	5/27 (18.5)	4/29	0.630
Serious adverse reaction (SAR)	0/39 (0.0)	0/39 (0.0)	0
Serious unexpected suspected adverse reaction (SUSAR)	0/39 (0.0)	0/39 (0.0)	0
Adverse events related to other study procedures	0/39 (0.0)	0/39 (0.0)	0
Serious adverse event (SAE)	1/39 (2.6)	0/39 (0.0)	1.000

Data are presented as n/N with data. Denominators for the self-reported side effects are based on the number of medication diaries that were returned: OAP 32/39 (82.1%), placebo 33/39 (84.6%). OAP, oral antibiotic prophylaxis

^a^Self-reported in medication diary during the three days of administration of study medication, including all participants who took at least one dose of study medication

To estimate whether our cohort was a representative sample of the patient population, we compared the baseline characteristics with a comparison cohort of 1597 patients. (Supplementary Table 5) Compared to the comparison cohort, the patients in the trial cohort were more often men, (67.9% versus 55.5%), had more colorectal malignancies (98.7% versus 74.5%), more minimally invasive procedures, but less abdominal surgery in the preceding year (3.8% versus 12.0%). The quality of life indicators, as shown in Table 2, all showed a more positive trend in the OAP group with a significant difference for social, physical, and emotional role functioning.

## Discussion

Due to the premature termination of this multicenter, double-blind, placebo-controlled randomized clinical trial, we were unable to determine the efficacy of OAP in terms of the risk of SSI and other postoperative complications.

The use of oral antibiotic prophylaxis in colorectal surgery is a controversial topic. Several studies demonstrated a reduced risk of SSI when OAP was administered before surgery [23, 24]. However, the question of whether preoperative oral antibiotic prophylaxis is effective without MBP remains unanswered as all RCTs published to date combine OAP with MBP. The best available evidence on OAP efficacy is provided by a recent network meta-analysis that aimed to study the best strategy for bowel preparation. This study also emphasized the knowledge gap on OAP without MBP, as the absence of RCTs that included this strategy as a treatment arm forced the authors to estimate the efficacy of OAP based on indirect comparisons only. Though based on indirect comparisons, a significant reduction in organ/space SSIs was found with OAP only, compared to no preparation (OR 0.13 [95% CrI 0.02–0.55]). This strategy was superior to combining OAP with MBP.

Data on the effectiveness of OAP without simultaneous MBP is also provided by several retrospective observational studies that compared the different bowel preparation strategies. These studies reported conflicting results on effectiveness [25–36]. Potential confounding by indication and limited numbers of patients treated with only OAP hamper concluding on the effectiveness of OAP in the absence of MBP and exemplifies the need for well-controlled and adequately powered studies.

We consider the randomized design as a major strength of our study, which facilitated the unbiased assessment of the efficacy of OAP and its potential drug-related side effects. Although the quality of our design is high, selective participation could not be prevented entirely. Unfortunately, not all potential participants were screened. Patients suffering from multiple or more severe comorbidities were not always considered for participation even though they were eligible. This might have had an impact on the generalizability of the study population. Also, multiple other studies were being conducted within this patient population, which competed with our inclusions. Baseline characteristics of our cohort showed potentially relevant differences with those from a historical cohort of patients undergoing colorectal surgery from a different hospital. There are indications that the patients that we included differed from the source population. For example, the percentage of patients with colorectal malignancy in our cohort was higher. A recently implemented national screening program for colorectal cancer led to the detection of malignancies in an earlier stage. In general, these patients are in a better clinical condition, and surgery is less radical, which lowers the risk of SSI.

Treatment with OAP was associated with a significant improvement in perception of quality of life at six months after surgery. At the same time, worsening was seen for patients treated with a placebo. In the absence of an effect of OAP on any of the clinical outcomes that could have been a possible explanation for this improvement, we suggest further investigation to study whether and how OAP might impact the quality of life.

Because of the small sample size, we were unable to study the safety of OAP thoroughly. However, several patients who received OAP reported mild gastrointestinal side effects and an unappealing taste. When OAP is considered for implementation in the future, patients should be informed about these potential side effects and the necessity of completing the entire three-day course of OAP despite these side effects. Another important safety concern is the risk of developing antibiotic resistance. We found the prevalence of colistin and tobramycin resistance at baseline to be 16.7 and 39.4%, respectively. The prevalence of carriage of tobramycin and colistin-resistant species did not increase in both treatment arms. We compared our findings with the results obtained with the implementation of selective decontamination of the digestive tract (SDD), a comparable antibiotic prophylaxis containing tobramycin, colistin, and nystatin that is used in several Dutch ICUs. In a post hoc analysis of two multicenter trials, it was shown that during SDD use, the prevalence of colistin resistance ranged from 1.7 to 2.8%, and of tobramycin resistance from 6.2 to 8.0%, respectively [37]. Other studies on SDD found a comparable prevalence [38–40]. The selective culture methods that we used in our study are known to have a higher sensitivity to detect antimicrobial-resistant Gram-negative bacteria [41], and may explain the higher prevalence observed compared to other studies. Due to the small number of patients, we were unable to exclude that OAP may increase the risk of developing antibiotic resistance.

### Ethical considerations

At the time this trial was initiated, there was no consensus within the Dutch surgical community on whether OAP should be used before colorectal surgery and, as a result, it was not part of clinical care in the vast majority of hospitals. Because of the uncertainty about the efficacy of the intervention, there was clinical equipoise regarding the use of OAP [42]. The shift started when the findings of a single-center before-after study were published. This before-after study was performed in the same setting without routine MBP administration [21]. In contrast to previous observational studies, the risk of confounding by indication was minimized because OAP was implemented as the standard of care and prescribed to all patients who underwent elective colorectal surgery. After implementation, a 42% reduction was observed in the risk of SSI and mortality within 30 days after surgery (aRR 0.58 [95% CI 0.40–0.79)]. Due to the single-center aspect of the study and the risk of residual confounding, a well-controlled study was deemed necessary to confirm the treatment effect.

We faced multiple problems recruiting participants throughout the entire study period despite our efforts to improve the inclusion rate. The unexpectedly low recruitment rate was communicated with the participating hospitals. Supported by the effectiveness found in the observational study, several investigators considered awaiting the trial results unacceptable and decided to implement OAP to reduce SSI rates. We decided to end the trial prematurely, because the assumption of clinical equipoise regarding the administration of OAP was no longer valid, and the use of a placebo was no longer ethically justifiable.

To conclude, we could not evaluate the efficacy of OAP on SSI risk and other postoperative complications after colorectal surgery due to premature termination of this double-blind, placebo-controlled, randomized clinical trial. Due to the loss of clinical equipoise, we will no longer consider the use of placebo in clinical trials on the efficacy of OAP ethics. Considering the current evidence, we recommend the implementation of OAP in clinical practice and the continued monitoring of infection rates and antimicrobial resistance.

## Supplementary information

**Additional file 1: Supplementary Table 1.** Participating hospitals. **Table 2.** Overview of study procedures and follow-up. **Table 3.** Overview of progress per study site. **Table 4**. Microorganisms cultured from three patients with SSI. **Table 5.** Comparison of characteristics with data from an observational cohort of elective colorectal surgery patients.

## Data Availability

The metadata file is available on the DataverseNL data repository https://dataverse.nl/dataset.xhtml?persistentId=hdl:10411/7BKGVB.

## Abbreviations

ASA: American Society of Anesthesiologists
BMI: Body mass index
CI: Confidence interval
DOT: Days on therapy
DSMB: Data safety and monitoring board
eCRF: Electronic case report form
ESBL: Extended-spectrum beta-lactamase
HRE: Highly-resistant Enterobacteriaceae
IQR: Interquartile range
GCP: Good clinical practice
ICU: Intensive care unit
MBP: Mechanical bowel preparation
OAP: Preoperative oral antibiotic prophylaxis
RCT: Randomized controlled trial
RR: Risk ratio
SAE: Serious adverse event
SAR: Serious adverse reaction
SDD: Selective decontamination of the digestive tract
SSI: Surgical site infection
SUSAR: Suspected unexpected serious adverse reaction
