# Supplementary material for: Prevention of severe infectious complications after colorectal surgery using oral non-absorbable antimicrobial prophylaxis: results of a multicenter randomized placebo-controlled clinical trial
Source: Antimicrob Resist Infect Control. 2020 Jun 15;9:84. doi: 10.1186/s13756-020-00745-2 (PMC7294517; doi:10.1186/s13756-020-00745-2)
Supplement: Supplementary file 1 — Additional file 1: Supplementary Table 1. Participating hospitals. Table 2. Overview of study procedures and follow-up. Table 3. Overview of progress per study site. Table 4. Microorganisms cultured from three patients with SSI. Table 5. Comparison of characteristics with data from an observational cohort of elective colorectal surgery patients. [file 13756_2020_745_MOESM1_ESM.docx]

**SUPPLEMENTARY MATERIAL**

| **Supplementary Table 1.** Participating hospitals | |
| --- | --- |
| **Hospital** | **Role in the study** |
| UMC Utrecht, Utrecht | Study coordination |
| Amphia Hospital, Breda | Distribution study medication  Analysis of rectal swabs |
| UMCG, Groningen | Inclusion of participants |
| Reinier de Graaf Gasthuis, Delft | Inclusion of participants |
| Meander Medical Center, Amersfoort | Inclusion of participants |
| St. Antonius Hospital, Nieuwegein | Inclusion of participants |
| Erasmus MC, Rotterdam | Inclusion of participants |
| Admiraal de Ruyter Ziekenhuis, Goes | Inclusion of participants |

| **Supplementary Table 2.** Overview of study procedures and follow-up | | | | | | |
| --- | --- | --- | --- | --- | --- | --- |
|  | **Study phase** | | | | | |
| **Study procedures** | **Screening** | **Enrollment** | **Intervention** | **Surgery** | **30 days after surgery** | **6 months after surgery** |
| Recruitment | ● |  |  |  |  |  |
| Eligibility screening |  | ● |  |  |  |  |
| Informed consent |  | ● |  |  |  |  |
| Allocation |  | ● |  |  |  |  |
| Rectal swab |  | ● |  |  | ● |  |
| Quality of life questionnaire |  | ● |  |  |  | ● |
| Intake study medication |  |  | ● |  |  |  |
| Self-report of side effects |  |  | ● |  |  |  |
| Report protocol related AE |  | ● | ● | ● | ● | ● |
| Report of SAE |  | ● | ● | ● | ● | ● |
| Report of SUSAR, SAR |  |  | ● | ● | ● | ● |
| Evaluation primary outcome |  |  |  |  | ● |  |
| Evaluation of secondary outcomes |  |  |  |  | ● | ● |
| ● Procedures performed by or with the participant ● Safety reporting ● Outcome assessment  AE, Adverse Events; SAE, Serious Adverse Events; SAR, Serious Adverse Reaction; SUSAR, Suspected Unexpected Serious Adverse Reaction | | | | | | |

| **Supplementary Table 3.** Overview of progress per study site | | | | | | | | | |
| --- | --- | --- | --- | --- | --- | --- | --- | --- | --- |
| **Hospital description** | | |  | **Study period** | | | | | |
| **Hospital** | **Category** | **Estimated No. eligible patients / yr** |  | **Study initiation** | **First recruitment** | **Last recruitment** | **Inclusion period, months** | **No. patients screened** | **No. patients included** |
| A | University | 60 |  | Mar 2017 | Apr 2017 | May 2018 | 14 | 28 | 13 |
| B | General | 150 |  | Jun 2017 | Jul 2017 | Aug 2018 | 14 | 34 | 16 |
| C | General | 200 |  | Jul 2017 | Sept 2017 | May 2018 | 9 | 8 | 6 |
| D | General | 250 |  | Aug 2017 | Sept 2017 | Aug 2018 | 12 | 54 | 33 |
| E | University | 130 |  | Dec 2017 | Jan 2018 | Mar 2018 | 3 | 4 | 3 |
| F | General | 150 |  | Dec 2017 | Apr 2018 | Jun 2018 | 3 | 7 | 7 |

| **Supplementary Table 4**. Microorganisms cultured from three patients with SSI | | | |
| --- | --- | --- | --- |
| **Type of SSI** | **Intervention** | **Tissue** | **Microorganisms cultured** |
| Deep incisional | OAP | Wound | *Pseudomonas aeruginosa*  *Enterococcus faecium*  *Enterococcus faecalis*  *Citrobacter freundii* |
| Organ/space | OAP | Abdominal pus | *Proteus mirabilis*  *Enterococcus faecium* |
| Organ/space | OAP | Abdominal pus | *Bacteroides ovatus*  *Actinomyces neuii* |
| OAP, oral antibiotic prophylaxis; SSI, surgical site infection | | | |

| **Supplementary table 5.** Comparison of characteristics with data from an observational cohort of elective colorectal surgery patients | | |
| --- | --- | --- |
| **Variable** | **PreCaution cohort**  **N = 78** | **Comparison cohort**  **N = 1,597** |
| Age in years, *median (IQR)* | 68 (61 – 73) | 68 (60 – 76) |
| Male sex | 53/78 (67.9) | 887/1,597 (55.5) |
| ASA classification |  |  |
| ≤2 | 56/74 (75.7) | 1,068/1,508 (70.8) |
| BMI in kg/m^3^, *median (IQR)* | 27 (23 – 29) | 25 (22 – 28) |
| Obese | 14/77 (18.2) | 240/1,570 (15.2) |
| Abdominal surgery in the previous year | 3/78 (3.8) | 192/1,597 (12.0) |
| Colorectal malignancy | 77/78 (98.7) | 1,190/1,597 (74.5) |
| Wound class |  |  |
| Clean contaminated (class 2) | 76/78 (97.4) | 1,422/1,597 (89.0) |
| Contaminated (class 3) | 2/78 (2.6) | 124/1,597 (7.8) |
| Dirty (class 4) | 0/78 (0.0) | 51/1,597 (3.2) |
| Type of resection |  |  |
| Right sided hemicolectomy | 22/78 (28.2) | 437/1,590 (27.5) |
| Left sided hemicolectomy | 6/78 (7.7) | 162/1,590 (10.2) |
| Sigmoid and rectum resection | 43/78 (55.1) | 675/1,590 (42.5) |
| Other | 7/78 (9.0) | 287/1,590 (18.1) |
| Surgical approach ^a^ |  |  |
| Open | 15/78 (19.2) | 797/1,590 (50.1) |
| Laparoscopic | 47/78 (60.3) | 565/1,590 (37.4) |
| Robotic laparoscopic | 16/78 (20.5) | 228/1,590 (14.3) |
| Perioperative intravenous antibiotic prophylaxis | 74/77 (96.1) | 1,513/1,582 (95.6) |
| Data are presented as n/N with data (%), or median (interquartile range). The comparison cohort consists of all patients who underwent elective colorectal surgery between 2012 and 2015 in the Amphia Hospital (Breda, the Netherlands). ASA, American Association of Anesthesiologists; BMI, body mass index; IQR, interquartile range  a. Laparoscopic procedures that were converted are classified as open procedures | | |
